# Supplementary material for: Vitamin D Modified DSS-Induced Colitis in Mice via STING Signaling Pathway
Source: Biology (Basel). 2025 Jun 18;14(6):715. doi: 10.3390/biology14060715 (PMC12190092; doi:10.3390/biology14060715)
Supplement: Supplementary file 1 [file biology-14-00715-s001.zip › Supplementary Materials S1: 16s-report of gut microbiota/src/doc/032615062393/032615062393.html]

客户常见问题的解释和描述

不同测序长度（v3-v4区/v4区/meta测宏基因组）的影响？

16S rRNA编码基因序列共有9个保守区和9个高可变区。其中，V4区其特异性好，数据库信息全，是细菌多样性分析注释的最佳选择。我们通过大量的测序试验证明用v4区扩增出菌群结果的可以很好的反应样本的菌群结构用于后续的数据建模分析。

原始数据fastq文件格式问题为什么打不开？

Fastq文件是用来存储碱基序列的原始数据，一般用于NCBI数据上传和用来做后续的生物信息分析的原始数据，fastq原始文件数据文件较大，打开文件可能导致 Windows 系统死机，建议使用性能较好的计算机或者使用更适合处理大量数据的 Unix/Linux 系统打开。如果要看数据分析结果，而不是用来自己处理数据进行分析的话，直接看分析结果报告就可以。

提供的Fastq文件有没有去除引物和barcode？

样本fastq文件中barcode去除了，引物没有去除

原始数据格式以及数据如何上传？

Fastq格式

原始fastq格式是一个文本格式用于存储生物序列（通常是核酸序列）和其测序对应的质量值。这些序列以及质量信息用ASCII字符标识。通常fastq文件中一个序列有4行信息：如

第一行：序列标识，以 @开头。格式自由，允许添加描述信息，描述信息以空格分开。

第二行：序列信息，不允许出现空格或制表符。一般是明确的DNA或RNA字符，通常大写

第三行：用于将序列信息和质量值分隔开。以 +开头，后边是描述信息或者不加。

第四行：质量值， 每个字符与第二行的碱基一一对应，按照一定规则转换为碱基质量得分。进而反映该碱基的错误率，因此字符数必须和第二行保持一致。

Fasta格式

fasta是一种基于文本用于表示核苷酸序列或氨基酸序列的格式。在这种格式中碱基对或氨基酸用单个字母来编码，且允许在序列前添加序列名及注释。由两部分信息组成：如

第一行：序列标记，以 >开头，接序列的标识符，序列标识符以空格结束，后接描述信息。为保证分析软件能区分每条序列，每个序列的标识必须具有唯一性。

第二行：序列信息，使用既定的核苷酸或氨基酸编码符号。

数据提交

原始数据（Raw data），常见的是illumina机器产生的fastq文件，这一类文件需要向NCBI的SRA数据库进行提交，SRA是NCBI为了并行测序的高通量数据（massively parallel sequencing）提供的存储平台。完整提交SRA需要一些独立项目的分步提交，包括BioProject、BioSample、Experiment、Run等，每一部分用以描述数据的不同属性。

如何判断测序质量是否合格？

原始的Tags数据会经过质控、过滤、去嵌合体，最终得到有效数据（Effective Tags）。所以在判断测序质量是否合格时应该从几个方面去判断。

打开文件reads\_length\_distribution.txt

报告里所有的txt打开如果格式不对的话，可以用excel表打开

表中统计了每个样本的原始序列数量：raw-tags，无完全匹配的单条序列数量：singleton，及其比例：singleton%，比对到最终ASVs的序列数量：tagsmatchedASVs，及其比例：tags-matched-ASVs%和每个样本的ASVs数量。

首先判断下机数据raw\_tags和匹配到ASVs的agsmatchedASVs的数据量是否满足测序要求，一般下机数据量达到3万条reads以上满足测序需要，谷禾16s样本的测序深度可以达到10万条reads左右。如果数据量不够则需要重新补测样本。通过观察无完全匹配的单条序列数量singleton及其比例singleton%，可以反应出有效序列的转化率，该比例越小序列的利用转化率就越高。

如何了解样品的主要菌群构成情况？

通过与数据库silva138进行比对，用Qiime2对代表序列进行注释，并分别在各个分类水平：domain（域），kingdom（界），phylum（门），class（纲），order（目），family（科），genus（属），species（种）统计各样本的群落组成。

Qiime2得到的物种注释结果

所有样本门、纲、目、科、属、种构成情况详见文件目录： 04\_Taxonomic/taxa\_plot/index.html

这一部分可以看到每个样本的物种构成比例，Taxonomic Level 可以选择Level1 ~ Level7 界门纲目科属种，不同分类水平下的物种构成。

以下为标准化到10万reads的物种构成结果文件，可以使用excel打开。

04\_Taxonomic/taxa\_plot/level-2.csv  ~  level-7.csv   分别是门到种水平

OTU/ASVs 表文件： 01\_pick\_otu/otu-table100k-tax.txt

以下为百分比的物种构成结果文件，可以使用excel打开。

04\_Taxonomic/relative/feature\_table\_L2.txt  ~  feature\_table\_L7.txt  分别是门到种水平greengene 13\_8版本物种注释结果文件 01\_pick\_otu/gg\_13-8table100k-tax.txt

利用对应数据作图，得到各分类水平主要菌群构成柱状图

taxanomyBar/taxon\_hist/without\_unknown/  不包含未命名物种

taxanomyBar/taxon\_hist/ include\_all/  包含未命名物种

top10/ 取含量最高的前10个物种作为整体做柱状图

all/  取前10个物种，将其他物种归为others做柱状图

如何了解分组内部的多个样本的重复性以及多样性情况？

观察分组内部多个样本的重复性如何可以从以下几个方面考虑

首先在各分类水平的柱状图的菌属构成来看

从构成图来看，Flu组和ZW3.7组，组内样本重复性较好。Ctrl组中Ctrl.2明显区别于组内另外两个样本，可以去掉该样本。而ZW3.8组内样本间差异性较大。

比如人体肠道或小鼠肠道样本本身个体差异性较大，菌群结构组成复杂，即便通过不同疾病的分类的样本，但营养饮食、代谢以及环境的影响都会改变肠道菌群的构成，所以有可能组内样本间差异性会比较大。而经过单因素处理的样本组内差异会比较小。

通过beta多样性分析PCA,PCoA,MNDS 也可以大致观察组内样本重复性情况，左图组内样本重复性较好，右图组内样本间差异性较大，两组间的区割不是很明显。

在加圈图的beta多样性分析中，右下角有给出PC1和PC2的P值，小于0.05则差异显著。

所以在前期实验设计时，尽量选择同一批次相同处理的小鼠或其他样本，避免组内差异的影响。并且要预留好多余的样本，假如组内重复样本太少，比如组内只有3个样本，如果去掉一个差异性较大的样本，一个分组内只有2个样本，会影响后续组间差异比较。并且在做组间差异性比较分析是每组重复样本至少要3个样本。

Alpha多样性是可以做针对单个样品中物种多样性的分析的判断，包括chao1指数、ace指数，shannon指数以及simpson指数等指数越大，说明样品中的物种越丰富。

其中chao指数和ACE指数反映样品中群落的丰富度（species richness），即简单指群落中物种的数量，而不考虑群落中每个物种的丰度情况。指数对应的稀释曲线还可以反映样品测序量是否足够。如果曲线趋于平缓或者达到平台期时也就可以认为测序深度已经基本覆盖到样品中所有的物种；反之，则表示样品中物种多样性较高，还存在较多未被测序检测到的物种。

而shannon指数以及simpson指数反映群落的多样性（species diversity），受样品群落中物种丰富度（species richness）和物种均匀度（species evenness）的影响。相同物种丰富度的情况下，群落中各物种具有越大的均匀度，则认为群落具有越大的多样性。

稀释曲线是利用已测得序列中已知的各种OTU的相对比例，来计算抽取n个（n小于测得Reads序列总数）Tags时各Alpha指数的期望值，然后根据一组n值（一般为一组小于总序列数的等差数列，本项目公差为500 ）与其相对应的Alpha指数的期望值绘制曲线。

对其中的几个样品不满意，如何去除重新作图？

若报告中存在样本重复性较差的样本，或数据质量较差、异常的样本，或者有其他分组或排列需求时，可以提供重新分析作图。需要注意的是，在后续做组间差异分析时，每个组内的样本是至少为3个。若对样本进行删减后低于这一标准则无法做相关分析。所以刚开始实验设计时要注意，分组内部尽量选用同一批次 重复性较好的样本，提前预留好充分的平行样。

不同的样本之间差异大吗？不同分组之间能否用菌群差异来区分？

观察不同分组间差异的大小可以观察随机森林分类效果图。

路径在diff\_analysis/RF

图中以该分类水平下选取用于区分不同分组间的差异性起到关键性影响因素的物种作为标志物作图。标志物按重要性从大到小排列，图中随机森林值error rate 表示用随机森林方法预测分组之间的错误率，分值越高代表所选取的标志物准确度不高，并不能很好的用于区分各分组，分组差异不显著。分值越低证明分组效果比较好。

上图中的随机森林按照门和属以及代谢途径分别进行分析作图，各自都有单独文件，报告中仅给出了一个图，其他文件需要到目录中查看。可能存在门或属区分效果不佳，但是代谢途径区分效果较好。

随机森林筛选出来的物种是用于区分所有分组的重要标志。分值越高代表该物种用于区分所有组之间的重要性越大。

不同分组之间的样品主要是在菌的数量？菌的构成？还是特定菌种？还是功能构成上有差异？

组间物种组成显著性差异分析根据不同组别的群落丰度数据，运用严格的统计学方法可以检测出组间微生物群落丰度差异的分类。通过群落结构对应的功能基因预测也可以用于区分分组之间的差异。

在OTU数量上比较多样性的差异可以看组间的箱型图：

对应数据Alpha\_diversity\_Boxplot.pdf  

从上到下分别是shannon，simpson和chao1，最上方标有统计检验的pvalue。

通过在所有组之间进行筛选差异物种做箱型图：

有门水平、属水平和功能上进行，比较

图中每一个箱型图，代表某一个物种在所有组之间比较构成显著差异

对应统计结果数据：

xxx.Groups.all.meanTests.csv

这个文件列出了该分类水平水平所有物种的统计结果

xxx.Groups.sig.meanTests.csv

这个文件列出了该分类水平具有统计学差异的物种的统计结果

例如这是一个表格的截图

红框  mean\_  是分组组间的平均值

蓝框  sd\_  代表组间的标准差

粉色  .test  代表不同统计检验结果的P-value P值，这里有var检验 T 检验 Wilcoxon检验

绿色  \_BH   例如Wilcoxon.test\_BH代表Wilcoxon.test检验BH矫正的Q-value，Q值

Alpha多样性指数分别代表什么？如何区分？这里提到shannon值越大，物种多样性越高。为什么有的文章提到，shannon值越大，物种多样性越低，哪个正确？

物种丰度

- chao1指数：评估样品中所含OTU的总数。其公式为：

Schao1：估计的OTU数量；  
Sobs：实际观察到的OTU数量；

n1：只含一条序列的OTU的数量；

n2：含两条序列的OTU的数量。

物种多样性

- Shannon指数：包含着种数和各种间个体分配的均匀性两个部分。如果每一个体都属于不同的种，多样性指数就最大；如果每一个体都属于同一种，则其多样性指数就最小。用来估算微生物群落的多样性，shannon值越大，物种多样性越高。其计算公式：

Sobs：实际观察到的OTU数量；

ni：第i条OTU的序列数量；

N：所有的序列数。

有的文章提到shannon值越大，物种多样性越低，则计算公式是1-

- Simpson指数：随机抽取的两个个体属于不同种的概率，Simpson指数越大，物种多样性越高。其计算公式：

Sobs：实际观察到的OTU数量；

ni：第i个OTU的序列数量；

N：所有的序列数。

Chao1的经典公式如

Sobs表示样本中观察到的物种数目。F1和F2分别表示singletons和doubletons的数目。

Chao1指数还有另外一种修正偏差的公式，在scikit-bio[1]上也有提到了(注：QIIME使用的是scikit-bio)，如下：

由经典公式可以看到，当doubletons为0（即F2为0）时计算的结果没有意义，修正公式可以解决这个问题。  
 

可以这样理解这个修正公式（虽然不太严格）：它从singletons中拿出1条来（严格来说与经典公式相比还不到1条），当作doubletons，这样分母一定会大于0。

从QIIME代码来看，QIIME调用skbio.diversity.alpha.chao1时bias\_corrected为默认值True，表示按经典公式计算。

测序深度

- Coverage：反应测序深度，goods\_coverage 指数越接近于1，说明测序深度已经基本覆盖到样品中所有的物种。其计算公式：

Cdepth：goods\_coverage指数表示测序深度；

n1：只有含一条序列的OTU数目；

N：为抽样中出现的总的序列数。

寻找组间菌群比较选取物种标志物（即组间差异物种），报告中给了几种方法： LEfSe基于线性判别分析（Linear discriminant analysis，LDA）的分析方法、 Anosim检验、随机森林分类树、STAMP 软件差异分析用于比较两个样本（Fisher’s exact test 分析）、两组样本（Welch’s t-test 分析）或多组样本 （ANOVA 分析）之间物种的丰度。以上方法区别是什么？我们选用哪种分析结果更可靠？

LEfSe基于线性判别分析（Linear discriminant analysis，LDA）的分析方法，其将线性判别分析与非参数的Kruskal-Wallis 以及Wilcoxon秩和检验相结合，从而筛选组与组之间生物标记物Biomarker，即组间差异显著物种。

组间物种差异盒形图是通过Kruskal-Wallis、Var检验和单因素方差分析one-way相结合，筛选出组间差异性物种。

随机森林分析使用R包“randomForest”默认设置比较组间差异。

Lefse分析选取分组差异的标记物种运用较为广泛

Lefse分析为什么有时候只分析了部分分组，或没有出图，是没有分析完全吗？

Lefse 分析是选取组间差异标记物，即用于区分该分组和其他各分组的优势物种，所以不一定所有分组都能找出优势物种，即组间差异标记物

Lefse分析是对所有分组进行比较的，若图中只显示了部分分组的差异结果，则说明lefse 分析在该分组情况下只在部分组中找到差异标记物，而其余分组没有差异标记物（即该分组没有找到相较于其他分组具有明显优势物种）

若该位置没有出图，或图片打不开，则说明在该分组情况下，lefse分析没有找出组间差异标记物。

进化分支图：位置Lefse\_Analysis/out\_formant.cladogram.png

LDA值分布柱状图：位置Lefse\_Analysis/out\_formant.png

特征表： Lefse\_Analysis/out\_formant.res

若对应位置没有图片或图片为空，则组间没有差异标记物

不同图的统计检验是怎么做的，代表什么意义？

LEfSe基于线性判别分析（Linear discriminant analysis，LDA）的分析方法，其将线性判别分析与非参数的Kruskal-Wallis 以及Wilcoxon秩和检验相结合，从而筛选组与组之间生物标记物Biomarker，即组间差异显著物种。

组间物种差异盒形图是通过Kruskal-Wallis、Var检验和单因素方差分析one-way相结合，筛选出组间差异性物种。绘制组间物种差异性箱型图。

随机森林分析使用R包“randomForest”默认设置比较组间差异。随机森林是机器学习算法的一种，它可以被看作是一个包含多个判定树的分类器。其输出的分类结果是由每棵判定树“投票”的结果。由于每棵树在构建过程中都采用了随机变量和随机抽样的方法，因此随机森林的分类结果具有较高的准确度，并且不需要“减枝”来减少过拟合现象。随机森林可以有效的对分组样品进行分类和预测。

Tukey主要应用于3组或以上的多重比较,适合于各组例数相等的每两两比较。Tukey检验的一个重要的优点是非常简单，而且所需实验样本相对较少。其检验结果的可信度达到95%置信水平时，最少的情况下只需6个样本进行验证（改善前3个样、改善后3个样）。

metagenomeSeq组间物种及功能差异分析是用R开发的一个包，其安装可以直接R CMD INSTALL metagenomeSeq\_1.12.0.tar.gz。 metagenomeSeq的基本思想，用normalization实现分类注释时的biases处理，同时用零膨胀高斯分布（zero-flated  Gaussian distribution）处理了测序深度所带来的影响，在此基础上，利用线性模型找到存在的差异所在。

LEfSe详解及相关问题答疑

LEfSe的定义

LEfSe分析即LDA Effect Size分析，是一种用于发现和解释高维度数据生物标识（基因、通路和分类单元等）的分析工具，可以进行两个或多个分组的比较，它强调统计意义和生物相关性，能够在组与组之间寻找具有统计学差异的生物标识（Biomarker）。

LEfSe结果分析

一般地，在微生物多样性分析结果中，会出现两个图，一张表（ LDA值分布柱状图、进化分支图及特征表）。

LDA值分布柱状图

这个条形图主要为我们展示了LDA score大于预设值的显著差异物种，即具有统计学差异的Biomaker，默认预设值为2.0（看横坐标，只有LDA值的绝对值大于2才会显示在图中）；柱状图的颜色代表各自的组别，长短代表的是LDA score，即不同组间显著差异物种的影响程度。

进化分支图：

小圆圈: 图中由内至外辐射的圆圈代表了由门至属的分类级别（最里面的那个黄圈圈是界）。不同分类级别上的每一个小圆圈代表该水平下的一个分类，小圆圈的直径大小代表了相对丰度的大小。

颜色: 无显著差异的物种统一着色为黄色，差异显著的物种 Biomarker跟随组别进行着色，红色节点表示在红色组别中起到重要作用的微生物类群，蓝色节点表示在蓝色组别中起到重要作用的微生物类群。未能在图中显示的Biomarker对应的物种名会展示在右侧，字母编号与图中对应（为了美观，右侧默认只显示门到科的差异物种）。

3. 特征表：

Lefse的统计结果特征表： Lefse\_Analysis/out\_formant.res

该文件可以拖到Excel表打开

第一列是样本中从门到属水平所有分类单位的列表

Lefse会逐一判断这些分类单位的在分组之间是否具有统计学显著性差异。

第二列：各组分丰度平均值中最大值的log10，如果平均丰度小于10的按照10来计算；

如果该分类单位为体现出显著组间差异，则后三列为空

对于具有统计学差异的分类单位

第三列：差异基因或物种富集平均丰度最高的分组组名；

第四列：LDA差异分析的对数得分值；

第五列：Kruskal-Wallis秩和检验的p值，若不是Biomarker用“-”表示。

默认LDA>2，P<0.05

通常根据第4列的LDA差异分析对数得分值和第五列的P值，可以描述组间具有显著差异的分类单位统计学效力强弱

所以这里这里LDA值分布柱状图上的物种都是P值小于0.05 具有统计学差异的

LEfSe分析原理

LEfSe分析中的检验

参数检验与非参数检验的区别

参数检验：即总体分布类型已知，用样本指标对总体参数进行推断或作假设检验的统计检验方法。

非参数检验：即不考虑总体分布类型是否已知，不比较总体参数，只比较总体分布的位置是否相同的统计方法。

参数检验分类：T检验，方差分析，（要求：方差齐性、正态分布）。

选用非参数检验的情况有：①总体分布不易确定（即不知道是不是正态分布）

②分布呈非正态而无适当的数据转换方法③等级资料等。

一般地，微生物多样性分析中，样本群落分布不确定，多采用非参数检验。

1. 秩和检验：

秩和检验是一种非参数检验法，它是一种用样本秩来代替样本值的检验法。根据样本分组的不同可分为两样本Wilcoxon秩和检验和多样本Kruskal-Wallis检验。

首先来了解几个容易搞混的词。

秩次（rank）：秩统计量，是指全部观察值按从小到大排列的位序；

秩和（rank sum）：同组秩次之和。秩和检验就是通过秩次的排序列求出秩和，进行假设检验。

a) Wilcoxon秩和检验（Wilcoxon rank sum test，也称为Mann-Whitney Test）：

基本思想是：若检验假设成立，则两组的秩和不应相差太大。通过编秩，用秩次代替原始数据信息来进行检验。

原理就是不管样本中的数据到底是多少，将两样本数据混合后从小到大排序，然后按顺序赋秩，最小的赋为1，最大的赋为n1+n2，分别对两个样本求平均秩，如果两个样本的平均秩相差不大，则说明两个总体不存在显著差异；反之，若相差较大，先分别求出两个样本的秩和，再计算检验统计量（含量较小的样本秩和）和统计量（期望秩和，查T值表可知）的P值并作出决策。

补充材料：Wilcoxon秩和检验是由F. Wilcoxon于1945年提出，1947年，Mann和Wiltney对Wilcoxon秩和检验进行了补充，后面就有了Mann-Wiltney检验。

b) Kruskal-Wallis秩和检验：

原理与两样本Wilcoxon检验类似。不同的是Kruskal-Wallis秩和检验针对多组独立样本，且进行的是H检验；在实际秩和与期望秩和差值的基础上计算检验统计量，最后计算出统计量的P值并作出决策。需注意的是，多组样本差异显著时，应进行多样本的两两比较的秩和检验。

2. LDA：

LDA的全称是Linear Discriminant Analysis（线性判别分析），是一种supervised learning（有监督学习）。有些资料上也称为是Fisher’s Linear Discriminant，由Ronald Fisher发明自1936年，是在目前机器学习、数据挖掘领域经典且热门的一个算法。

LDA的思想可以用一句话概括，就是“投影后类内方差最小，类间方差最大”。简单来说就是一种投影，是将一个高维的点投影到一个低维空间，我们希望映射之后，不同类别之间的距离越远越好，同一类别之中的距离越近越好。

PC3 括号里 的NA是什么意思？

PC1到PC3后的括号中有一个百分比。这个百分比代表这个变量可以解释的总体方差的百分比。NA就是占的总体方差的信息量极少，可以忽略不计。

通过一系列的特征值和特征向量进行排序后，选择主要排在前几位的特征值，PCoA 可以找到距离矩阵中最主要的坐标 “  如何知道排序后PC1、PC2、PC3分别是什么菌？我们想知道我们的分析结果中PC1、PC2、PC3分别指向什么菌？

PCoA是一种研究数据相似性或差异性的可视化方法，应用方差分解，对多维数据进行降维，从而提取出数据中最主要的元素和结构的方法。通过一系列的特征值和特征向量进行排序后，选择主要排在前几位的特征值，这里特征向量并不具体特值具体物种。

质控怎么判断？如果以chimeras%低于1来作为标准，某些样本大于1如何处理？还是按照二代测序有个质量Q值对应？稀释曲线应该只能说明测序数量足够大，但是不能判断测序质量高吧？

质控主要看测序原始质量，Q30比例。这一步在数据前处理分样和合并的时候就过滤了。还有chimeras比例，chimeras会在处理中剔除。之后的数据看稀释曲线，如果前期处理后稀释曲线不合格表明符合质量的测序数量不足。

韦恩图是用OTU来绘制的。不同OTU可能对应同一个物种，是否要用注释后的物种来做韦恩图比较合理呢？

OTU能对应到物种的注释比例不是很高，如果用物种会导致大量OTU归为无注释。

随机森林分类树中 “Error rate”:  表示使用下方的特征进行随机森林方法预测分类的错误率，越高表示基于菌属特征分类准确度 不高，可能分组之间菌属特征不明显。“ Error rate多少才算高，行业内是否有标准？

这个主要看最终ROC的结果，一般ROC大于0.7表明可以区分，大于0.85为较好效果，大于0.95以上为非常理想。error rate和ROC差不多。

随机森林的ROC曲线，每种颜色曲线是代表什么？ 曲线上的每一个点是什么意思？如何通过ROC曲线判断分组效果的情况？

ROC 曲线指受试者工作特征曲线 (receiver operating characteristic curve), 是反映敏感性和特异性连续变量的综合指针，通过构图法揭示敏感性和特异性的相互关系。ROC曲线每个颜色代表一个分组。ROC 曲线下的面积值在 1.0 和 0.5 之间。在 AUC>0.5 的情况下，AUC 越接近于 1，说明诊断效果越好。AUC在 0.5~0.7 时有较低准确性，AUC 在 0.7~0.9 时有一定准确性，AUC 在 0.9 以上时有较高准确性。AUC=0.5 时，说明诊断方法完全不起作用，无诊断价值。AUC<0.5 不符合真实情况，在实际中极少出现。在 ROC 曲线上，最靠近坐标图左上方的点为敏感性和特异性均较高的临界值。小于0.5的基本上模型无法有效区分分组，和随机差不多，UC<0.5 不符合真实情况，在实际中极少出现。有效值的一般要0.65以上。

ROC 曲线上，最靠近坐标图左上方的点为敏感性和特异性均较高的临界值。

ROC曲线图用于判断诊断的准确性。 （属水平）ROC曲线图诊断标准是什么？

ROC使用的是randomforest方法，该方法有特征重要性预测，会根据特征均属对模型准确性的提升来评估选择的特征。与LeFse不一定相同。

KEGGpathway 、COG、KO都是功能分析，它们有什么区别？能不能找出代谢功能对应的物种？

COG构成在组间存在显著差异的功能分类

通过KEGG代谢途径的预测差异分析，我们可以了解到不同分组的样品之间在微生物群落的功能基因在代谢途径上的差异，以及变化的高低。为我们了解群落样本的环境适应变化的代谢过程提供一种简便快捷的方法。

ko基因的差异分析图，除了能对大的基因功能分类和代谢途径进行预测外，我们还能提供精细的功能基因的数量和构成的预测，以及进行样本间以及组间的差异分析，并给出具有统计意义和置信区间的分析结果。

这里是根据菌群关系预测的功能分析，没有对应的物种关系

STAMP 软件load 文件报错，unknown parsing error，如何解决？

这个一般是文件或软件路径中有中文，需要全英文路径

E:\report\_16s\a项目\2021.03.19-report-16s-zdq10\10\_STAMP

例如在这个文件路径中，带有中文，导入数据后就会报错

档铺网——在线文档免费处理
